# P-1023. Unveiling the Clinical Consequences of Hypervirulent Clostridioides difficile RT181: First Spanish Outbreak Analysis

**DOI:** 10.1093/ofid/ofaf695.1219

**Published:** 2026-01-11

**Authors:** Tatiana Mata Forte, Carmen Palacios Clar, Lucia Ruiz Salazar, Juan Jose Reyes Lujan, Miriam Estébanez, German Ramirez Olivencia, Paula Pescador Martin, Fco Javier Membrillo de Novales

**Affiliations:** Hospital Gomez Ulla, Madrid, Madrid, Spain; Hospital Universitario Doctor José Molina Orosa, SANTA CRUZ TENERIFE, Canarias, Spain; Hospital Gomez Ulla, Madrid, Madrid, Spain; Hospital Gomez Ulla, Madrid, Madrid, Spain; Hospital Central de la Defensa Gómez Ulla, Madrid, Madrid, Spain; Hospital Gomez Ulla, Madrid, Madrid, Spain; Hospital Gomez Ulla, Madrid, Madrid, Spain; Hospital Gomez Ulla, Madrid, Madrid, Spain

## Abstract

**Background:**

*Clostridioides difficile* infection (CDI) is a growing healthcare concern, intensified by the emergence of hypervirulent strains that increase severity, recurrence, and mortality. While ribotype 027 is well documented, other strains such as ribotype 181 (RT181) have been predominant in regions like northern Greece but had not been previously reported in Spain. This study describes the first outbreak of RT181 in a Spanish hospital and evaluates its clinical impact.

**Table 1.**
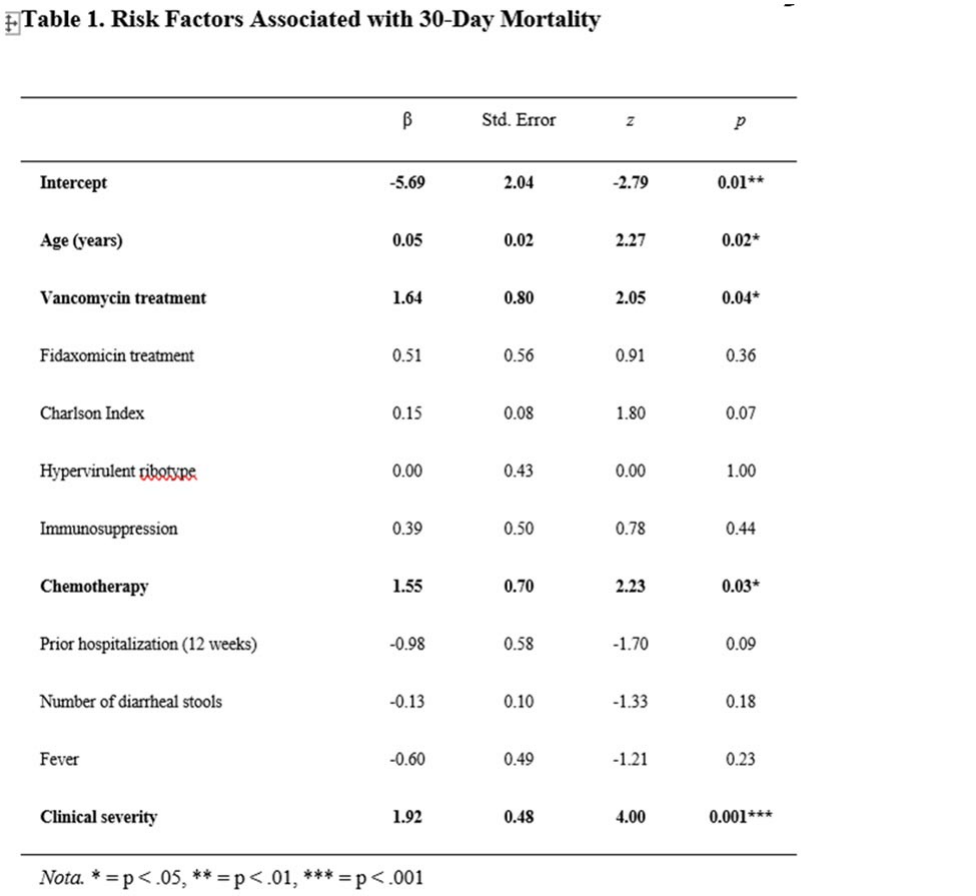
Risk Factors Associated with 30-Day Mortality

**Table 2.**
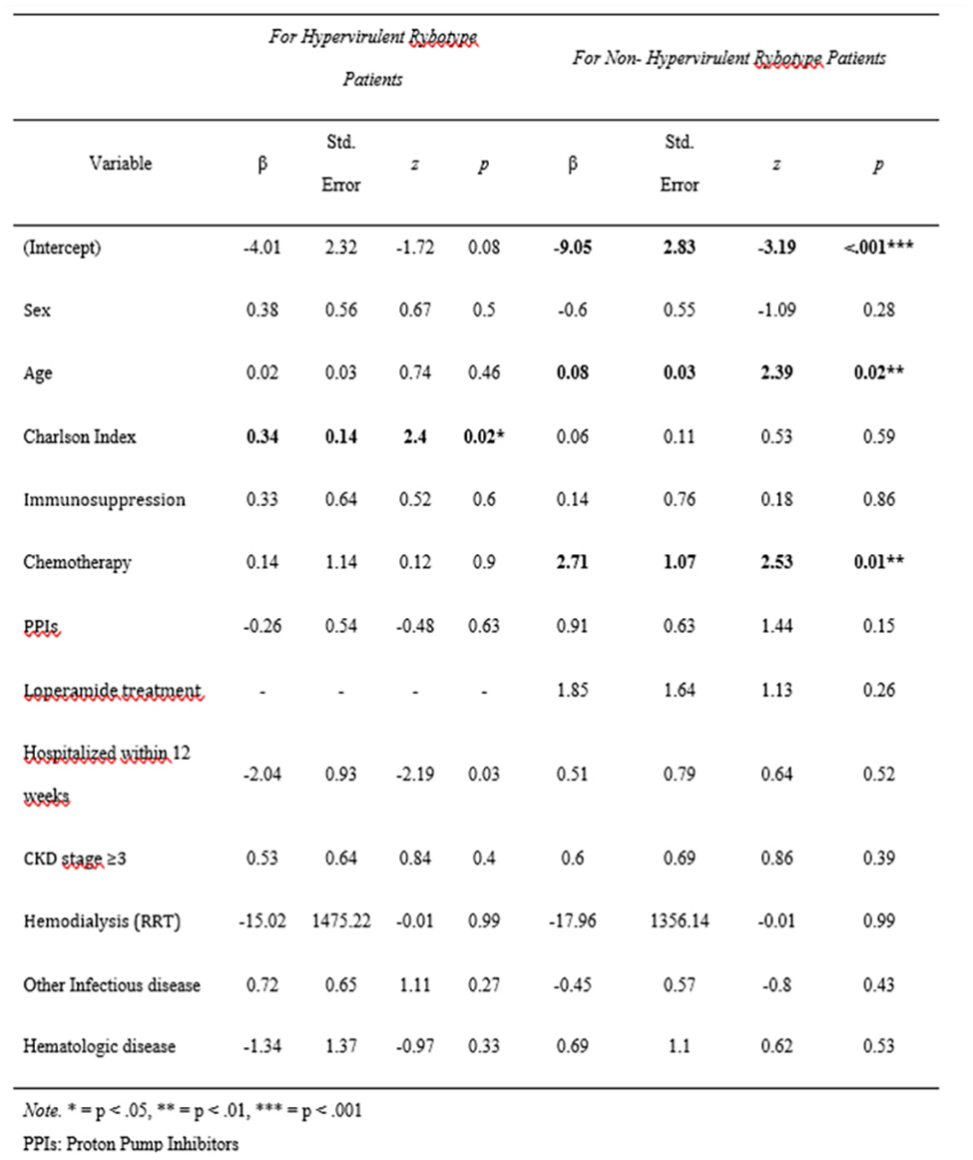
Risk Factors Associated with Mortality by Ribotype (Hypervirulent vs Non-Hypervirulent)

**Methods:**

A retrospective observational study was conducted on all PCR confirmed CDI cases of a single center in Madrid, Spain, during a CDI outbreak between December 2022 and December 2023. Diagnosis included PCR detection of toxin A/B genes and capillary PCR ribotyping for RT181. Data collected included demographics, Charlson Index, clinical severity, prior antibiotic use, treatment regimens, 90-day recurrence, and 30-day mortality. Statistical analysis compared descriptive statistics and multivariate logistic regression.

**Table 3.**
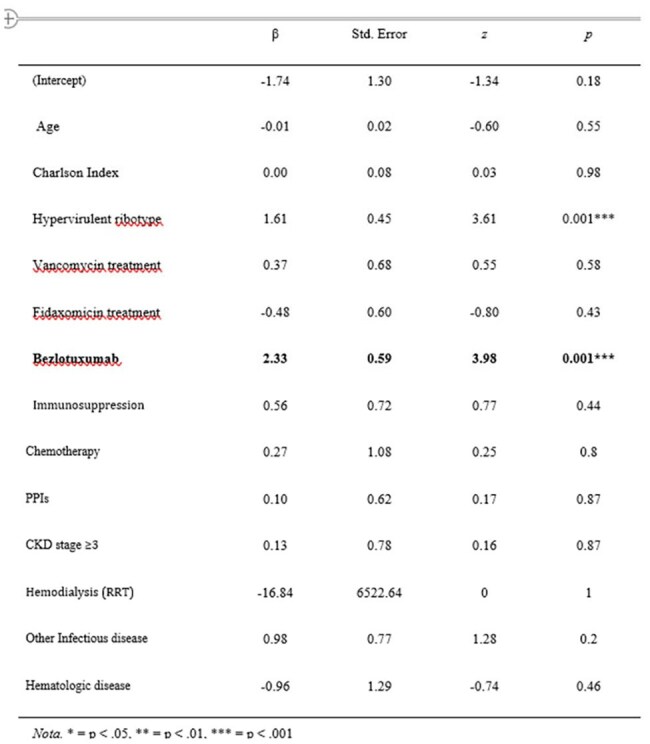
Risk Factors Associated with Recurrence

**Table 4.**
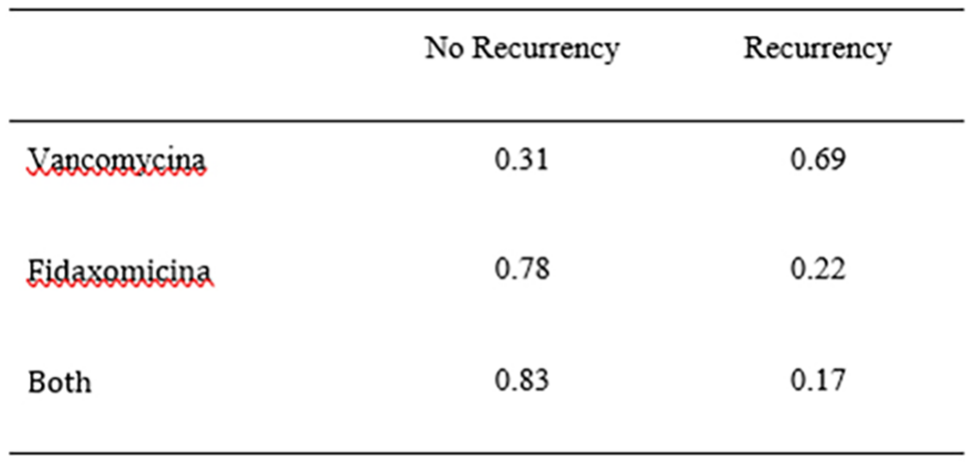
Recurrence Rates According to Treatment

**Results:**

Among 251 CDI cases, 108 (43.2%) were attributed to RT181. The mean patient age was 79 years, with 53.2% male and a high prevalence of comorbidities. Severe CDI occurred in 11.6% and fulminant colitis in 2.4%. Continued antibiotic therapy was required in 18.8% of cases. Treatments included fidaxomicin (60.4%), vancomycin (39.6%), metronidazole (5.6%), and bezlotoxumab (16.4%). RT181 infection was associated with a higher recurrence rate (26.9%), particularly among those treated with vancomycin (69%) compared to fidaxomicin (22%) or combination therapy (17%). Thirty-day mortality was 28%, mainly influenced by advanced age, clinical severity and chemotherapy. Patients treated with vancomycin had a higher death rate than those treated with fidaxomicin. Hypervirulent infection was independently associated with female sex and predicted recurrence, while chemotherapy was a significant predictor of mortality only in non-hypervirulent cases. Regression analysis linked hypervirulent ribotypes to prolonged recovery.

**Conclusion:**

RT181 demonstrated hypervirulent behavior, with high recurrence and severe outcomes. Mortality was mainly driven by host factors. Vancomycin treatment was associated with an increase in mortality.

**Disclosures:**

All Authors: No reported disclosures

